# One Health ethics in context: Justice, interdependence, and normative pluralism

**DOI:** 10.1016/j.onehlt.2026.101467

**Published:** 2026-06-08

**Authors:** Andrea Ford, Lisa Boden, Paulo Ferrinho, Saana Jukola, Lucy Robertson, Jakob Zinsstag

**Affiliations:** a Centre for Biomedicine, Self and Society, The University of Edinburgh, Usher Building, 5–7 Little France Road, Edinburgh EH16 4UX, UK; b University of Edinburgh, Royal (Dick) School of Veterinary Studies, Easter Bush Campus, Roslin EH25 9RG, UK; c Global Health and Tropical Medicine, Instituto de Higiene e Medicina Tropical, Universidade Nova de Lisboa, 100 Rua da Junqueira, Alcantara, Lisbon, Portugal; d Philosophy Section, University of Twente, Drienerlolaan 5, 7522 NB Enschede, the Netherlands; e Norwegian University of Life Sciences, Faculty of Veterinary Medicine, Department of Paraclinical Sciences, Elizabeth Stephansens vei 15, 1433 Ås, Norway; f Swiss Tropical and Public Health Institute, Kreuzstr. 2, 4123 Allschwil, Switzerland; g University of Basel, Petersplatz 1, 4003 Basel, Switzerland

**Keywords:** Ethics, Critical bioethics, Relational ethics, Justice-oriented health frameworks, Contextual moral reasoning

Engaging with One Health issues invites a discussion of ethics – that is, the moral principles that govern actions – because One Health's radical and transformative aspects pose challenges to conventional ethical premises. For example, scholars have recently argued that attention to planetary boundaries and ecological health are not only essential to safeguarding human health, but require redesigning bioethics so that it is “less confined by anthropocentrism” and incorporating the biosphere in a way that “require[s] a new level of ethical imagination to achieve real world impact” [1]. One Health implies expanding the moral circle to include animals and ecological entities, leading to dilemmas about how to ethically conduct activities such as animal husbandry, wildlife management, or mining. Moreover, One Health calls for a change in how to deal with ethical tensions themselves.

For example, there have been calls for a more rigorous ethical framework centered around justice [2], which would expand beyond the problematic “balancing of interests” found in rights-based discourse to account for the inevitable conflicts that arise. Situations in which the One Health approach is applied, such as combating zoonoses and addressing human food insecurity, often entail conflicts between the interests of different human stakeholders (e.g. public health institutions, farmers, the food industry) – but moreover, they entail conflicts between values, since values shape human perceptions of the ethical care that different entities warrant and how they should be in relationship with each other. One Health ethics must not only extend beyond humans, but beyond individual, rights-holding subjects of moral worth whose interests are understood to compete.

## 1 Engaging with diverse frameworks of justice

To address these tensions meaningfully, One Health ethics must move beyond academic theory and engage directly with grounded frameworks of justice. Most importantly, this includes social movements and practical theorisations of social justice, including environmental justice (how different people's material surroundings are cared for), reproductive justice (whose reproduction is valued and supported), and epistemic justice (how knowledge is made and used). We have elaborated upon this elsewhere [3]^1^:

“The people at the forefront of this work are often Indigenous communities, disadvantaged communities, those experiencing disabilities or neurodivergence, and Black, Brown, and ethnically and geographically minoritised peoples – in short, the majority of the world's population and those who suffer disproportionately from injustices. Crucially, ethical reasoning and addressing ‘vulnerabilities’ should not be done out of context, in a top-down or universalising way – rather, the complex ethical issues raised by One Health need to be addressed in partnership with, and perhaps led by, marginalised groups. One Health is well placed in this respect due to its practice of engaging with local communities… Such collaboration requires both a priori informed consent and a nuanced view of the ethics of knowledge production, including considerations of who owns knowledge and how downstream benefits are distributed.”

Existing frameworks for integrating diverse perspectives could be usefully adopted. For example, the concept of ‘Two-Eyed Seeing’ advocates for the importance and feasibility of combining Indigenous and Western ways of knowing. It aims to use the best of both worldviews to aid understanding, solve problems, and promote ethical exchanges.

## 2 Justice and rights

Justice frameworks typically expand upon and in some ways fundamentally diverge from more conventional paradigms based on rights. Rights are usually atomizing, meaning that they are framed as belonging to individuals, whether human, animal, or otherwise (including individual species or populations). They are applied to entities rather than relationships or processes. This is because rights discourse evolved from an individualizing framework of what exists and can be known (in philosophical terms, an ontology and epistemology). Rights discourse is, therefore, “difficult to practically integrate with a recognition of interdependence among humans and other entities, in which rights conflict with each other and responsibilities must be equally central” [3].

Yet, rights are incredibly important. The framework of rights has advanced the wellbeing and liberation of many over the centuries, and remains powerful in the cultural imagination. ‘One Rights’ has been developed as a complementary approach to One Health that extends rights to animals. A legal framework for ‘One Health: the human-animal relationship’ has been proposed based on existing European laws. In certain contexts, such as legal dealings and advocacy initiatives, this language can prove pragmatic and effective. There have been efforts to establish legal protections for aspects of the environment, including recent documents from the European Court of Human Rights on climate protection, and work advocating the (human) right to a clean environment. Ecuador recognized “rights of nature” in its 2008 Constitution.

While the discourse of rights has offered important legal and moral tools, and in some instances continues to do so, its limitations become evident when addressing relational and systemic interdependencies that exceed the scope of individual entitlements. Hence, we hesitate to rely on it to frame One Health ethics. As we argue elsewhere, an approach to non-human animals, nature, or the environment that treats these as distinct from humans is not as ethically robust and transformative as an approach that sees humans, non-human animals, and their environments as mutually enmeshed ecosystems [3]. While extending rights to animals or nature does present a serious challenge to anthropocentric definitions of rights, which can be a pragmatic stepping stone that makes use of existing frameworks, it is inherently limited. Justice frameworks tend to centre relationships instead of rights. Some Indigenous and Black scholar-activists refer to “right relations” as an ethical guiding principle along these lines.

## 3 Normative pluralism and power

If we start with the premise that ethical judgments must be context dependent, One Health ethics needs to acknowledge and accept a diversity of ways to do “the right thing” instead of seeking universal judgments and guidelines. This is known as normative pluralism. Across contexts, norms and strategies for livelihood, animal welfare, ecosystem protection, and health will differ and require different ways of achieving consensus.

A clear example of complex ethical tensions in One Health is the widespread practice of culling animals during zoonotic disease outbreaks. Although it is a common method to prevent the spread of infectious pathogens, culling raises questions about how human safety should be balanced against animal welfare in agriculture and wildlife management.

The justification for culling and other measures to prevent diseases depends on normative assumptions – that is, value-based ideas about what is morally important. In the anthropocentric tradition, culling can be ethically justified on grounds of minimizing harm to humans. However, One Health ethics compel us to recognize that non-human beings and socio-ecological systems have more than an instrumental value and that harm to them has ethical bearing. This is related to a more comprehensive critique: the case for abolishing animal farming entirely, which can be defended on both animal rights and environmental grounds, and indeed has been articulated within a One Health approach.

However, normative positions either prioritising humans above other beings or elevating the status of animals above their conventional utilitarian or sentimental position must be submitted to societal scrutiny in a given context. As we have pointed out:

“Imposing an external framework may be inappropriate, and indeed might compromise the opportunity for conventional power-holding entities to learn from alternative ethical arrangements. For example, abolishing animal farming is an out-of-context argument when it comes to Indigenous foodways. Consider the North American example of the Suquamish people's relationship with salmon-fishing and whale-hunting, which is based on interdependence. Encouraging vegetarianism or veganism can have overtones of coloniality, enforcing ethical norms where they may not belong and promoting food hegemony instead of supporting food sovereignty” [3].

Certainly, there is a necessary distinction between large-scale industrial animal farming (the Western agricultural model) and more pastoral and localized forms of animal husbandry, which do not map neatly on to regions or communities. Industrial animal farming is being hegemonically exported all over the world from the USA and Europe, as part of contemporary food coloniality. It is important to distinguish between Western diets heavy with animal-source foods and more occasional consumption of animals; again, the former is now being exported and replicated. Vegetarianism and veganism can be understood as protesting against these industrial, Western norms, not against eating animals per se. There are many ways to consider ethical behaviour towards animals – for instance, “leaving them alone” might be seen as abandonment, or autonomy and respect. The point is that these ethical positions must be contextualized in historical and political relationships.

Ethical concepts and norms are not static or universal, but evolve in relationships shaped by power. To return to the case of the rights of nature, scholars have argued that it universalizes colonial modes of existence as natural, but also have recognized how the concept hybridizes distinct ways of knowing that have come into contact for historical reasons – in this case, de-colonial critiques of extractivism, sustainable development discourses about biodiversity, and indigenous struggles for territorial autonomy. Having sensitivity to history and power dynamics is essential to a One Health ethics that can accommodate different needs, positions, incentives, and visions of a just future.

## 4 Conclusion

To be fit for purpose in our complex world, One Health ethics must allow for diverse ways of knowing and acting morally to be taken seriously. It must be able to account for power and difference without flattening context or offering simplified solutions. And so, in this piece, we cannot offer a definitive position on One Health ethics, but instead gesture towards ethical engagements attuned to complexity, vulnerability, and justice. The field of One Health should embrace this challenge, not as a limitation, but as an opportunity to deepen its transformative potential.

## CRediT authorship contribution statement

**Andrea Ford:** Writing – review & editing, Writing – original draft, Project administration, Conceptualization. **Lisa Boden:** Writing – review & editing, Conceptualization. **Paulo Ferrinho:** Writing – review & editing, Conceptualization. **Saana Jukola:** Writing – review & editing, Conceptualization. **Lucy Robertson:** Writing – review & editing, Conceptualization. **Jakob Zinsstag:** Writing – review & editing, Conceptualization.

## Funding

This research was funded in whole, or in part, by The Wellcome Trust [Grant number 209519/Z/23/Z]. For the purpose of open access, the author has applied for a CC BY public copyright license to any Author Accepted Manuscript version arising from this submission.

## Declaration of competing interest

The authors declare the following financial interests/personal relationships which may be considered as potential competing interests:

Andrea Ford reports financial support was provided by Wellcome Trust. If there are other authors, they declare that they have no known competing financial interests or personal relationships that could have appeared to influence the work reported in this paper.

## Data Availability

No data was used for the research described in the article.

## Footnotes

1 Due to citation limits in this format, we have only cited work that we quote here. Fuller citations can be found in our report (SAPEA 2024).
